# Correction: Gold nanoparticles supported on supramolecular ionic liquid grafted graphene: a bifunctional catalyst for the selective aerobic oxidation of alcohols

**DOI:** 10.1039/d2ra90026b

**Published:** 2022-03-24

**Authors:** Mojtaba Mahyari, Ahmad Shaabani, Yasamin Bide

**Affiliations:** Faculty of Chemistry, Department of Polymer, Shahid Beheshti University G. C., P. O. Box 1983963113 Tehran Iran a-shaabani@sbu.ac.ir

## Abstract

Correction for ‘Gold nanoparticles supported on supramolecular ionic liquid grafted graphene: a bifunctional catalyst for the selective aerobic oxidation of alcohols’ by Mojtaba Mahyari *et al.*, *RSC Adv.*, 2013, **3**, 22509–22517, DOI: 10.1039/C3RA44696D.

The authors regret that Fig. 6A was re-used from an article published in the *Journal of Power Sources*^1^ without being cited and without permission from the publisher to reproduce the image.

The authors have now received the permission to reuse the image and the corrected caption is shown below:

Fig. 6 HR-TEM images of graphene oxide (A), and Au NP@SIL-*g*-G (B). (a). Reproduced from Hadi Hosseini *et al.*, with permission from Elsevier, 2014.

The Royal Society of Chemistry apologises for these errors and any consequent inconvenience to authors and readers.

## Supplementary Material
